# Identification of Selected Antibiotic Resistance Genes in Two Different Wastewater Treatment Plant Systems in Poland: A Preliminary Study

**DOI:** 10.3390/molecules25122851

**Published:** 2020-06-20

**Authors:** Magdalena Pazda, Magda Rybicka, Stefan Stolte, Krzysztof Piotr Bielawski, Piotr Stepnowski, Jolanta Kumirska, Daniel Wolecki, Ewa Mulkiewicz

**Affiliations:** 1 Department of Environmental Analysis, Faculty of Chemistry, University of Gdansk, Wita Stwosza 63, 80-308 Gdansk, Poland; magdalena.pazda@phdstud.ug.edu.pl (M.P.); jolanta.kumirska@ug.edu.pl (J.K.); daniel.wolecki@phdstud.ug.edu.pl (D.W.); ewa.mulkiewicz@ug.edu.pl (E.M.); 2 Department of Molecular Diagnostics, Intercollegiate Faculty of Biotechnology, University of Gdansk and Medical University of Gdansk, Abrahama 58, 80-307 Gdansk, Poland; magda.rybicka@biotech.ug.edu.pl (M.R.); krzysztof.bielawski@biotech.ug.edu.pl (K.P.B.); 3 Institute of Water Chemistry, Technische Universität Dresden, Bergstraße 66, 01062 Dresden, Germany; stefan.stolte@tu-dresden.de

**Keywords:** antibiotic resistance genes (ARGs), antibiotic-resistant bacteria (ARB), wastewater treatment plants (WWTPs), activated sludge (AS), constructed wetlands (CWs), environmental pollution, spread of resistance, tetracyclines, sulfonamides

## Abstract

Antibiotic resistance is a growing problem worldwide. The emergence and rapid spread of antibiotic resistance determinants have led to an increasing concern about the potential environmental and public health endangering. Wastewater treatment plants (WWTPs) play an important role in this phenomenon since antibacterial drugs introduced into wastewater can exert a selection pressure on antibiotic-resistant bacteria (ARB) and antibiotic resistance genes (ARGs). Therefore, WWTPs are perceived as the main sources of antibiotics, ARB and ARG spread in various environmental components. Furthermore, technological processes used in WWTPs and its exploitation conditions may influence the effectiveness of antibiotic resistance determinants’ elimination. The main aim of the present study was to compare the occurrence of selected tetracycline and sulfonamide resistance genes in raw influent and final effluent samples from two WWTPs different in terms of size and applied biological wastewater treatment processes (conventional activated sludge (AS)-based and combining a conventional AS-based method with constructed wetlands (CWs)). All 13 selected ARGs were detected in raw influent and final effluent samples from both WWTPs. Significant ARG enrichment, especially for *tet*(*B*, *K*, *L*, *O*) and *sulIII* genes, was observed in conventional WWTP. The obtained data did not show a clear trend in seasonal fluctuations in the abundance of selected resistance genes in wastewaters.

## 1. Introduction

Antibiotic resistance is arguably one of the greatest threats and challenges to public health and contemporary medicine worldwide. As a consequence of the widespread use of antimicrobials for human, veterinary and agricultural purposes, the number of antibiotic-resistant bacteria (ARB) is constantly increasing [1]. According to the published data, in 2015 in the European Union (EU) and European Economic Activity countries, ARB caused 671,689 infections and led to over 33,000 deaths [2]. Furthermore, the cost of hospitalisation of European patients infected by selected multidrug-resistant bacteria was estimated at least EUR 1.5 billion annually [3], while in the United States this cost was even higher and estimated at approximately USD 2.2 billion [4].

Antimicrobial compounds such as pharmaceuticals are classified to a group of contaminants of emerging concern (CECs) [5]. The results of many studies confirmed the presence of trace amounts of numerous antibiotics not only in municipal, hospital or industrial wastewater, but also in surface, ground and drinking water, as well as in soil and bottom sediments [6]. For this reason, and due to the growing awareness that the occurrence of these compounds in the environment may pose a threat to human health and ecological systems, the EU Commission has included three macrolide antibiotics (azithromycin, clarithromycin, erythromycin) (EU Decision, 2015/495 of 20 March 2015) as well as amoxicillin and ciprofloxacin (EU Decision, 2018/840 of 5 June 2018) in the European Union Watch List as substances subjected to monitoring. A comprehensive risk assessment of antimicrobial compounds in the environment for human health is complicated, but the risk is clearly recognized when concentrations sufficient for selection of resistant pathogenic strains are reached. Available data suggest that such minimum selective concentrations may be very low and, even at environmentally relevant antibiotic concentrations, the maintenance and selection of resistant bacteria can occur [7].

Antibiotic resistance in healthcare-associated infections are a common and alarming problem in many countries [8]; e.g., in Poland, where the incidence of these types of infections appear higher than in neighbouring countries [9]. There are several ongoing activities across countries to reduce rising antibiotic resistance problem. The European Surveillance of Antimicrobial Consumption (ESAC) network, and more recently the European Centre for Disease Prevention and Control (ECDC) [10,11] and the World Health Organization (WHO) Europe [12], have researched antibiotic utilisation across Europe including Poland. The available data indicate that Poland has one of the highest rates of total consumption of antibiotics among European countries [13]. Infections in Polish patients demonstrate the relationship between utilisation and resistance levels, i.e., the high sulfamethoxazole (SMX) consumption [14] accompanied by high levels of resistance to it [15]. Concerns with current antibiotic resistance rates in Poland are confirmed by data on severe *Acinetobacter baumannii* infections characterised by the high level of resistance to commonly used antibiotics [16,17], as well as data on MRSA epidemiology both in hospitalised adult patients, as well as new-borns with very low birth weight [18].

It has been confirmed that wastewater treatment plants (WWTPs) are significant reservoirs of antibiotic resistance genes (ARGs) and sources of their spread in the environment [19,20]. Due to the unregulated consumption of antibiotics, excretion by humans and animals or improper medication disposal methods, native forms of these compounds, their metabolites and transformation products end up in hospital and municipal wastewater. According to the published data, tetracyclines are present in WWTPs at concentration levels of 1300 ng/L, measured in raw wastewater [21] and even 1420 ng/L in effluent [22]. Sulfamethoxazole, the most often identified sulfonamide in WWTPs, was detected at concentration levels of 5597 ng/L in influent [23] and up to 6000 ng/L in treated wastewater [24]. The occurrence of SMX and its main derivative N^4^-acetylsulfamethoxazole in raw wastewater (1464 and 1763 ng/L, respectively) and effluent (508 and 16 ng/L, respectively) samples collected in WWTP1 were also reported [25]. In the environment, antibiotics are not only chemical pollutants that can exert toxic effects, but they are above all able to cause selection pressure [26]. WWTPs are considered probable hotspots for antibiotic resistance dissemination in the environment. The presence of antibiotic residues, even at low concentrations, combined with the high density and diversity of microorganisms (including pathogenic, commensal, environmental and indigenous) sustained by a nutrient rich environment, might facilitate the ARB proliferation and ARGs horizontal gene transfer (HGT) mediated by mobile genetic elements (MGEs), such as plasmids, transposons, integrons and bacteriophages [27]. Furthermore, WWTPs have a substantial impact on the spread and abundance of ARGs in the environment [28]. The data available in the literature indicate a significant increase in numbers of antibiotic resistance determinants in effluent discharged from WWTPs [1,29,30]. Different tetracycline resistance genes, e.g., *tet*(*A*, *B*, *C*, *G*, *L*, *M*, *O*, *Q*, *X*), as well as sulfonamide resistance genes, e.g., *sulI*, *sulII*, *sulIII* have been detected in the effluent of WWTPs [31]. The abovementioned data and results for the other ARGs confirm that effluent discharged from WWTPs is one of the major anthropogenic sources of these pollutants in the environment and can pose a real threat of spreading resistance to bacterial pathogens [19,32].

Since conventional activated sludge (AS)-based WWTPs seem to be inefficient in ARG removal, the implementation of additional wastewater cleaning processes is necessary. It has been demonstrated that conventional disinfectants and advanced oxidation processes, or their combinations, are not capable of significantly reducing the amount of ARGs. The membrane bioreactor technology and photocatalytic ozonation seem to be good technological solutions for the future; however, the possibility of their application in full-scale WWTPs, due to high costs, is questionable [33,34]. Constructed wetlands (CWs) have been suggested as a cost-effective, ecological and efficient technology in wastewater treatment. The removal mechanism of contaminants in CWs is complicated and consists of physical, chemical and biological processes among plants, substrates and microorganisms, which can be also affected by CW type, substrate type and plants [35,36,37,38,39]. CWs have been proposed as a promising alternative solution for removing a wide variety of conventional pollutants as well as antibiotics, and even ARB and ARGs [35,40]. The reduction of antibiotics and ARGs in CWs, as demonstrated, could be achieved at relatively similar or even higher rates than in conventional WWTPs [36]. On the other hand, it was also suggested that, although CWs effectively remove antibiotics, they probably stimulate the spread of ARGs [41].

Considering the fact that the abundance of ARGs in final effluent can be influenced by the type of WWTP technological processes and their operating conditions, the main aim of this research was to compare the occurrence and abundance of selected tetracycline and sulfonamide resistance genes before and after the purification process in two different in size and applied wastewater treatment processes (conventional AS-based and combining a conventional biological AS-based method with CWs) WWTPs. To examine possible seasonal fluctuations in gene abundances, the samples were collected over four seasons in 2018. A molecular-based approach and quantitative polymerase chain reaction (qPCR) technique were used to study ARGs in the WWTPs. The genes selected for the study represent different mechanisms of resistance such as: ribosome protection (coded by genes *tet*(*M*, *O*, *Q*)), efflux pump (coded by genes *tet*(*A*, *B*, *C*, *G*, *K*, *L*)), drug modification (coded by gene *tetX*) [42] and target modification (coded by *sul* genes) [43]. Moreover, when choosing genes, their location on different genetic elements (especially on MGEs which significantly influences the ARG dissemination) was taken into account. Tetracycline resistance genes are located on plasmids (*tet*(*A*, *C*, *K*, *O*)), transposons (*tet*(*B*, *M*, *X*) and chromosome (*tetQ*). In addition, some genes are found both on the integrons and chromosome (*tetG*), on plasmids and chromosome (*tetL*) or on transposons and plasmids (*sul* genes) [43,44].

It is well known that the nucleic acid extraction process and quality of isolated DNA are crucial for the subsequent polymerase chain reaction (PCR) gene detection in environmental samples and can influence the research results and its interpretation. Therefore, to select the optimal method for the isolation and purification of total DNA from WWTP samples, 10 commercially available DNA purification kits were tested and assessed in terms of the efficiency of isolation, DNA purity and the suitability of isolated DNA as template for PCR.

To analyze the data from qPCR, a relative quantification method was applied. The method is used in situations when the determination of the absolute copy number of the transcript is not necessary and reporting the relative change in gene expression is sufficient. In that case, the comparative C_T_ method (also known as ΔΔC_T_, or 2^-ΔΔCT^) is used to calculate relative changes in gene expression determined from qPCR experiments. The data are presented as the fold change in gene expression (normalized to 16S rRNA reference gene) between effluent and influent.

## 2. Results and Discussion

### 2.1. Evaluation of Total DNA Extraction Kits

The results of the total amount and quality of DNA for all tested DNA isolation and purification kits are presented in Table 1. The highest amount of DNA was obtained using the FastDNA SPIN Kit for Soil (kit numbered 4); however, the DNA sample isolated by this method did not equal the quality requirements; the determined A_260/230_ ratio indicated a significant content of contaminants, like residues of reagents used in the extraction process. It was found that DNA of a desired purity (A_260/230_ ≥ 1.8) was obtained only for the tested kits numbered 1, 2, 5, 6, 8, 9, and 10. In addition, the usefulness of all isolated DNA samples as a template for PCR was verified; PCR using the BACT 1369F and PROK 1492R primers (16S rRNA gene) and agarose gel electrophoresis were carried out. The specific 146 bp DNA fragment was identified in all PCR reactions tested (data not presented). The DNA extraction kits selected for the test were also evaluated according to the convenience of their use, time of the process and the cost of a single isolation. Considering all the above criteria, the GeneMATRIX SOIL DNA Purification Kit was selected as the optimal method for extraction and purification of total DNA from WWTP samples, and used in the further part of this study.

The selection of the appropriate procedure of genetic material isolation and purification at an early stage of the study is an important step for research based on PCR methods. DNA quality and purity significantly affect the course of reaction. The adequate method should be primarily selected for the type of extracted DNA and the specificity of sample matrix. To our knowledge, commercial kits designed especially for DNA isolation from wastewater or AS samples are not available, hence why kits for soil or fecal samples are used in this type of research [45,46,47,48]. WWTP samples as a multi-component research material require an individual approach in choosing the optimal method of DNA extraction. This type of sample contains complex polymers, organic matter, and numerous inorganic compounds—potentially enzymatic inhibitors that can be co-extracted with DNA. On the other hand, differences in the cell wall and membrane structures of microorganisms, as well as the susceptibility of different microorganisms to the cell lysis, significantly affects the efficiency of the extraction process. Our research confirmed that only half of the tested protocols achieve a satisfactory efficiency of the isolation process and receive a purified DNA sample without enzymatic reaction inhibitors, suitable as a template for PCR. The DNA extraction methods should be evaluated not only in terms of the quantity and purity of the obtained genetic material, but above all in terms of how faithfully the diversity of the sequences extracted reflects the structure of the microbial population in the studied environmental sample. Zielińska et al. [49] evaluated the usefulness for metagenomic sequencing of 7 out of the 10 DNA isolation and purification kits tested in our study. The high number of good quality reads, which made it possible to classify all of the obtained sequences at phylum level, high values of Shannon and Simpson indexes, which suggest a high level of the species diversity as well as low level of error rate among replicates, were observed for the GeneMATRIX SOIL DNA Purification Kit (EURx) (kit ID C3 in Zielińska et al. [49]). According to Morgan et al. [50], the reproducibility of extraction kit replicates is of high importance when tracking changes in microbial composition over time, between environments, with respect to seasonal and ecological changes. Moreover, results of the rarefication analysis of the obtained data revealed that the sampling of microbial communities is close to being complete for the analyzed kit [49].

### 2.2. Occurrence and Removal of ARGs in WWTPs

In this study, the role of selected WWTPs in the dissemination of ARGs in the environment have been investigated using a comparative qPCR method. Culture- or molecular-based approaches are followed to study the antibiotic resistance problem in WWTPs, each of them exhibiting some advantages and drawbacks. Culture-based methods are key to understand phenotypic characteristics of isolates and their resistance patterns, but they have limits with environmental bacteria (as the culturable fraction is only 1% of the total). Molecular methods—based on the isolation of the total DNA from the analyzed samples (influent, effluent or activated sludge) and the detection of specific nucleotide sequences coding ARGs using PCR and/or qPCR techniques—are applied to identify specific DNA targets in microorganisms that cannot be grown in the laboratory, or multiply very slowly but significantly contribute to the resistance problem [33].

**Figure 1:**
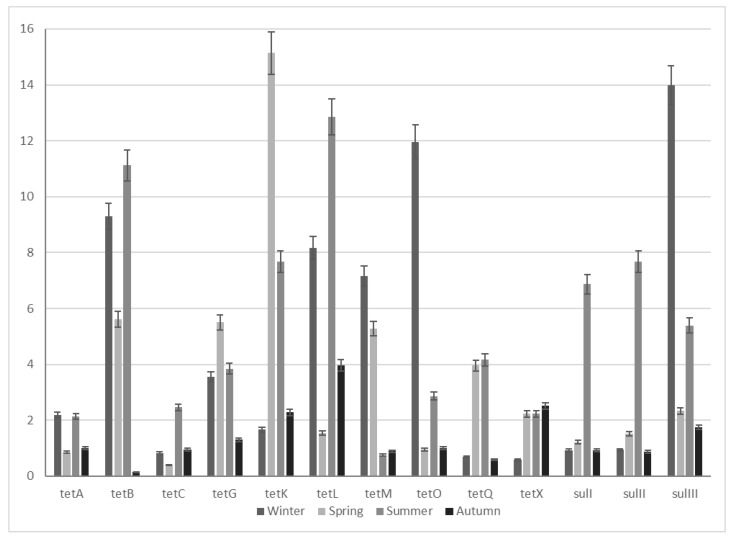
Enrichment of selected ARGs in the WWTP1 samples.

**Figure 2:**
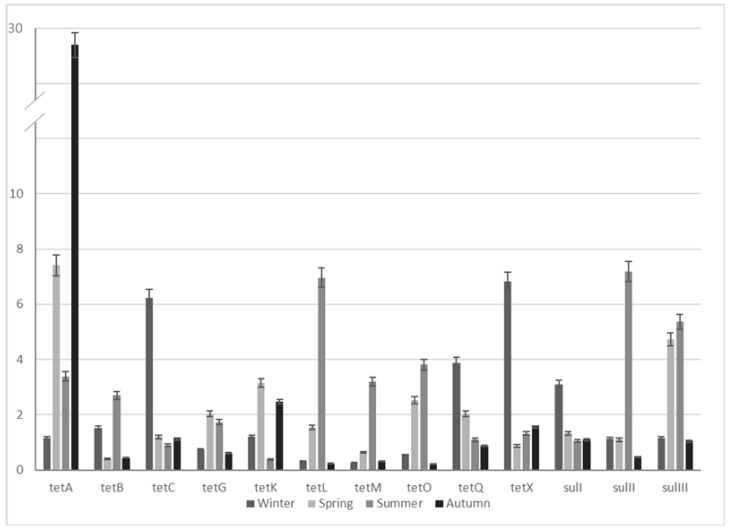
Enrichment of selected ARGs in the WWTP2 samples.

All 13 selected genes coding resistance to tetracyclines and sulfonamides were detected in raw influent and final effluent samples from both WWTPs. The results of the resistance gene enrichment obtained for WWTP1 are presented in Table 2 and in Figure 1. Generally, an increase of the ARG abundance from the influent to the effluent has been observed. The most significant enrichment (more than 10-fold) was recorded for *tet*(*B*, *K*, *L*, *O*) and *sulIII* genes. The corresponding data for WWTP2 are shown in Table 3 and in Figure 2. The obtained results indicate a tendency of the enrichment of selected ARGs occurring after the wastewater treatment process; however, these values are usually lower compared to the conventional WWTP1.

The obtained results may arise from the difference in size of compared WWTPs, which means that the average daily wastewater flow in WWTP1 is over 20 times higher than in WWTP2. It can be expected that a higher wastewater flow is associated with a higher load of antibiotic contaminants flowing through WWTP, which may promote selective pressure and ARB proliferation. In addition, even a small inflow of hospital wastewater to WWTP1 may be of key importance for the ARG content, due to the significantly higher concentration of antibiotics, ARB and ARGs compared to domestic wastewater. Therefore, the specially targeted separate treatment of hospital wastewater before discharge to conventional WWTPs is an issue that needs to be addressed by adapting to local circumstances.

An enrichment of ARGs after the conventional AS-based treatment process observed in this study is in agreement with the results reported by other researchers [1,30,51,52,53,54]. This could be explained by the selective conditions in WWTPs that may favor bacteria harboring resistance genes, ARGs or/and HGT between bacteria. The genes, for which the highest enrichment after the wastewater treatment process was observed (*tet(B, K, L, O),* and *sulIII*) in this study, are located on MGEs. These segments of DNA play an important role in adaptation process and are a means to transfer genetic information among and within bacterial species, which may cause their extensive prevalence [55]. On the other hand, some studies reported no change or even a decrease in the relative number of ARGs in the effluent after the conventional treatment process [56,57]. It seems that WWTPs with conventional treatment processes are not efficient in ARG removal. The wastewater treatment processes, which were primarily designed to remove nitrogen, biodegradable organic compounds, ammonia, nitrate, and phosphate are not effective in elimination of microbiological contaminants [33]. The available data from the literature show that biological processes may positively affect the ARB spread and selection as well as the ARG transfer [54,58]. The persistent selective pressure from antibiotic residues at sub-inhibitory concentrations, as well as the high density and diversity of microorganisms (including pathogenic, commensal, environmental and indigenous) sustained by a nutrient-rich environment, create favourable conditions for antibiotic resistance dissemination [27]. Moreover, WWTP sludge was recognized as the main source of tetracycline- and sulfonamide-resistant bacteria and genes discharged into the water environment. A significant enrichment of *tet* (*B, G, H, S, T, X*) and *sul* (*I, II*) genes in relation to 16S rRNA was observed [59]. In the face of the observed ARG enrichment in the wastewater treatment process, Mao et al. [59] emphasize the need for an improved understanding of how to manipulate WWTP operational variables, e.g., by decreasing the nutrient-to-microorganism ratio and thus limiting energy sources for ARB and as a consequence of metabolic deficiencies inhibiting resistance plasmid replication and promoting the loss of antibiotic resistance. According to Biswal et al. [51], the key factors affecting the prevalence of antibiotic resistance determinants are the operational parameters of WWTPs such as the residence times of hydraulics and solids, which can affect the dynamics of genetic material exchange and the distribution of ARGs in bacteria passing through the system. It was found that the activated sludge process did not affect the removal of ARG-carrying *Escherichia coli*, but increased the abundance of multiple ARGs in the bacterial genome. In turn, it was observed that the physicochemical systems were capable of removing ARB at a high rate, hence the bacterial density (potential ARG donors for HGT) was several orders of magnitude lower, which would significantly reduce the rate of ARG transfers compared to systems with AS. The authors suggested that observed differences in ARG dynamics for the two wastewater treatment types would be the result of the balance between the effectiveness of ARB removal and the HGT rates [51].

Although the quantitative analysis of ARGs in WWTP1 samples has not been studied before, the studies of antibiotic resistance of fecal coliforms and enterococci based on culture methods have been conducted by other authors [25,58,60]. Tetracycline-resistant isolates were detected in all sampling points, including influent and effluent samples, of the WWTP1. It was found that 20% of enterococci and 23% of *E. coli* isolates were resistant to tetracyclines [58]. In turn, resistance to SMX was observed in 11% of *E. coli* isolates. It should be also noted that 75% of SMX-resistant *E. coli* were simultaneously resistant to tetracyclines. The frequency of sulfonamide resistance genes (*sul I–III*) detected in *E. coli* strains of wastewater origin was similar as in other environmental compartments, including clinical ones. The molecular analysis of genes responsible for resistance to sulfonamides among SMX-resistant *E. coli* isolates showed a prevalence of *sulII* (81%) and *sulI* (50%) genes. Moreover, 31% of isolates simultaneously carried both *sul* genes. The *sulIII* gene was rarely detected—it was found in 6% of SMX-resistant isolates from the WWTP effluent [25]. The research concerned not only the analysis of antimicrobial resistance patterns in isolates from wastewater samples, but also on the presence of integrons, which are associated with antibiotic resistance dissemination phenomenon. Class 1 and 2 integrons were detected in 32% and 3% of antibiotic-resistant *E. coli* isolates, respectively, and were related to an increase of resistance to selected antimicrobial agents and multidrug resistance [60]. The positive selection of bacteria with resistance patterns in wastewater processes based on AS was observed. Resistance rates noted for bacteria isolated from treated wastewater was higher than that observed in corresponding influent. The treatment process favored both tetracycline- [58] and SMX-resistant [60] bacteria.

It has been confirmed that the application of CWs allowed a significant increase in the removal of antibiotics and other medicines from the wastewater [34,61,62,63,64,65,66]. CWs have been also shown to be more efficient in the removal of ARGs than conventional WWTPs [36,38,61,62,63,64]. Nolvak et al. [61] reported a decrease in the proportions of different ARGs (including *tet(A*, *B*, *M),* and *sulI*) in effluent (compared to the influent) during the treatment process in horizontal subsurface flow CWs. Moreover, the observed removal efficiency for *sulI* gene was better than in conventional WWTPs [61]. Liu et al. [62] found that the total absolute abundances of *tet* genes and 16S rRNA were reduced by 50% in wastewater using CWs. Significantly reduced absolute abundances of ARGs (*tet(O*, *M*, *W*, *A*, *X),* and *intI1*) and 16S rRNA were also reported by Huang et al. [64]. In another study, the removal efficiencies of 16S rRNA, *intI1* and *tet* genes among four different CW treatment systems ranged from 33% to 99% [38]. The observed differences in 16S rRNA in influent and effluent confirmed that studied CWs were capable of bacterial removal from wastewater; however, an increase in the relative abundance of ARGs indicated a risk of the release of relatively more antibiotic-resistant bacteria in proportion to total bacteria into environment [38,62,64]. The results obtained in our study also showed an enrichment of selected ARGs. These values are generally lower compared to the results obtained for the conventional WWTP system which might suggest that the introduction of an additional plant-based purification step increases the efficiency of removing ARGs; however, the amount of ARGs was still higher than in the raw influent. It has been suggested that the ability of the treatment systems to filter out bacteria contributes greatly to the reduction of ARB and, therefore, ARGs from wastewater in CWs (especially those with vertical flow) [38]. Sorption and biological processes occurring in CWs have been proposed as key mechanisms influencing the fate of ARGs during wastewater treatment. Biological processes can both lead to ARG transmission and proliferation as well as cause their degradation [36,62]. When being removed from wastewater, ARGs with their host bacteria were partially deposited in CWs, especially in surface soil which provided optimal conditions for their survival and development. Environmental stressors such as antibiotics and heavy metals accumulated in surface soil could promote horizontal gene transfer between foreign and indigenous bacteria [38]. Other authors suggested [67] that the survival of rhizospheric bacteria of CWs can be also associated with their increased resistance to various environment stressors.

Some authors observed the relationship between ARG reduction efficiency and CW flow type. It was reported that CWs with subsurface flow removed ARGs more effectively [61] compared to CWs with surface flow [68]. The filtration capacity of subsurface flow CWs for bacteria is higher than that of surface flow CWs [38]. In addition, the removal efficiency of horizontal subsurface flow CWs for ARGs was higher (over 50%) than that of vertical wetlands, especially for *sul* genes [37].

The reports available in the literature suggest that, just like conventional WWTPs, CWs could be considered as hotspots for the spread of antibiotic resistance in the environment. Song et al. [69] evaluated the fate of ARGs (*sul* and *tet*) in three lab-scale vertical flow CWs. They found out a positive correlation between abundances of ARGs and the accumulation of SMX and tetracyclines in different layers of CW substrate. Positive correlations were also observed between the abundance of *tet* genes and the antibiotic concentration in the effluent. Although the effluent had lower abundances of ARGs than that in the wetland media, the occurrence of ARGs in effluent might still pose risk for public health. Moreover, the relative abundances of *sul* and *tet* genes showed a significant increase in all samples during the SMX and tetracycline treatment period [69].

### 2.3. Seasonal ARG Changes

The analysis of obtained results did not show clear trend in seasonal fluctuations in abundance of selected tetracycline and sulfonamide resistance genes in wastewater. However, for conventional WWTP1, enrichment of most studied ARGs was observed in the summer, and to a lesser extent in spring and winter seasons. On the other hand, WWTP2 enrichment was more frequently noted only in summer and spring months. The explanation of ARB and ARG seasonal fate is not clear so far, and the conclusions from numerous studies regarding seasonal fluctuations of antibiotic resistance determinants in WWTP systems are often divergent [70,71,72]. While some studies showed higher release loads of ARB and ARGs in wastewater samples in spring and summer seasons than in winter months, other authors indicated an increase in numbers of tetracycline, sulfonamide, and vancomycin resistance genes in winter [73]. There are also studies that did not confirm the obvious seasonal fluctuations in the occurrence of ARGs detected in WWTP systems [74,75], which is consistent with the results obtained in this study. The reasons for the quantitative fluctuations of antibiotic resistance determinants in different seasons are complicated and depend on many factors, which include the variability in antibiotic consumption, the microbial composition variation in the wastewater and AS, and the presence of antimicrobial residues in wastewater or the co-selection of heavy metal resistance [73].

## 3. Materials and Methods

### 3.1. Characterization of Wastewater Treatment Plants

Two full-scale municipal WWTPs located in northern and central Poland were investigated. Conventional AS-based WWTP1—“Municipal Wastewater Treatment Plant Gdańsk Wschód” collects domestic wastewater from the population of about 570,000 people in the area of Gdańsk, Sopot, Pruszcz Gdański, Żukowo, Kolbudy and in a small part from local industry (5%), as well as hospital wastewater (0.17%). The daily average wastewater flow is 96,000 m^3^ with a 24-h retention time. Mechanical treatment units in this WWTP consist of mechanical screens, aerated sand traps with grease removal traps and radial-flow primary sedimentation tanks. Biological treatment units consist of 6 multiphase MUCT reactors (typical UCT system additionally equipped with a transitional chamber which can optionally serve as a nitrification or denitrification chamber and with deaeration chamber, where the mixture of treated wastewater and AS, recirculated from nitrification chamber to denitrification chamber, is de-oxidized) and 12 radial-flow secondary sedimentation tanks. The plant is also equipped with fermenter, which discharge pre-fermented sludge to sewage before primary sedimentation tanks and thus increases the effectiveness of biological phosphorylation [76]. The effluent from WWTP1 is transported via a pipeline into the Bay of Gdańsk and discharged 2.3 km away from the coastline. WWTP2, “Municipal Wastewater Treatment Plant in Sochaczew”, described in detail by Wolecki et al. [66], represents a system which combines the method of biological wastewater treatment with AS and constructed wetlands. CWs have been implemented in the second stage of wastewater treatment (biological). Contact between the plants and wastewater (mixed with AS) occurs only in the rhyzophytic zone. The following plant species were used in CWs culture: European spindle (*Euonymus europaeus*), Grey willow (*Salix cinerea*), Papyrus (*Cyperus papyrus*), Reed (*Phragmites australis*), Rushes (*Juncus tenageia* *Ehrh.*), Spathiphyllum (*Spathiphyllum Adans.*), Summer lilac (*Buddleja davidii* *Franch*), Sweet flag (*Acorus calamus*), Yellow iris *(Iris pseudacorus*) and Yellow pimpernel (*Lysimachia nemorum*). CW plants are placed in greenhouse with the total area of 1835.6 m^2^, where strict conditions—an optimal air humidity and temperature (35–38 °C)—are maintained for appropriate plant growth. WWTP2 is comparatively smaller than WWTP1, with an average wastewater flow of 4470 m^3^ per day. The wastewater collection from the area of Sochaczew city concerns domestic inflow from approximately 37,000 residents. The effluent from WWTP2 is discharged to Utrata River. The average values of the main WWTP1 and WWTP2 technological parameters: biological and chemical oxygen demand, total nitrogen and phosphorus, as well as total suspended solids from the sampling period, are presented in Table 4.

### 3.2. Samples Collection and Preparation

Samples of raw influent and final effluent from both studied WWTPs for quantitative analysis of ARGs were collected in January, April, July and October 2018. Additionally, to develop the optimal method of DNA purification, AS samples from the aeration chamber of the biological reactor of WWTP1 were collected in July 2018. Wastewater and AS samples were collected into sterile bottles, transported to the laboratory and stored at 4 °C.

Influent (400 mL) and effluent (2 L) samples were filtered using 1.2-μm glass microfiber filters (VWR, Leuven, France) to remove considerable size contaminants, and next through 0.22-μm mixed cellulose esters membrane filters (Merck Millipore, Cork, Ireland), to retain all biological material. Obtained filters were cut into smaller pieces, transferred to the 15-mL screw cap centrifuge tubes with 6 mL of 1 × PBS and shaken for 20 min (1000 rpm/min) at room temperature. Then the filters were transferred to clean tubes and the procedure was repeated with the new portion of PBS. Both suspensions were combined and centrifuged for 10 min (8000× *g*). All obtained precipitates were stored at −20 °C.

### 3.3. Selection of the Optimal DNA Isolation and Purification Method

In order to select the optimal method for isolation and purification of total DNA from WWTPs samples, 10 commercially available DNA purification kits (Table 1), dedicated to soil or fecal samples, were tested. The representative research material was AS, which was chosen as the most complex WWTP matrix, due to a significant content of exopolysaccharides and adsorbed on the surface of sludge biopolymers, organic matter and numerous inorganic compounds. AS samples (20 mL) were placed in 50-mL screw cap centrifuge tubes and centrifuged for 10 min (8000× *g*). Obtained precipitate was used for DNA purification procedures performed according to the manufacturer’s protocols. All purified DNA samples were subjected to spectrophotometric analyzes using Colibri Microvolume Spectrometer (Titertek Berthold, Pforzheim, Germany) to determine the concentration and purity of DNA (A_260/280_ and A_260/230_ ratios). To ascertain the suitability of isolated DNA as a template for standard PCR, a reaction using the BACT 1369F and PROK 1492R primers (16S rRNA gene) was carried out (primers sequences are listed in Table 5). All reactions were performed in 50 μL volumes in a T100™ Thermal Cycler (Bio-Rad, Hercules, CA, USA) and contained 80 ng of template DNA, 0.4 mM dNTPs (A&A Biotechnology, Gdynia, Poland), 0.5 µM of each primer (Genomed, Warsaw, Poland) and 0.75 units of Marathon DNA polymerase in 1 × Marathon PCR buffer (A&A Biotechnology, Gdynia, Poland). The cycling profile included: 94 °C for 5 min, then 30 cycles of 94 °C for 30 s, 56 °C for 30 s, 72 °C for 30 s, and a final step of 72 °C for 5 min. The presence of PCR product (146 bp in length) was confirmed using agarose gel electrophoresis. 2.0% BASICA LE Agarose gels (Basica Prona™ Agarose, ABO, Gdansk, Poland) were prepared in 1 × TBE buffer, and visualized after staining with ethidium bromide.

### 3.4. Isolation and Purification of Total DNA in Influent and Effluent Samples

Samples were prepared according to the procedure described in Section 3.2 and DNA purification procedures were performed using GeneMATRIX SOIL DNA Purification Kit (EURx, Gdańsk, Poland) according to the manufacturer’s protocols. All purified DNA samples were subjected to spectrophotometric analyses using Colibri Microvolume Spectrometer (Titertek Berthold, Pforzheim, Germany) to determine the concentration and purity of DNA (A_260/280_ and A_260/230_ ratios). Isolated DNA samples were stored at −20 °C.

### 3.5. Quantitative PCR

Quantitative PCR was used to analyze selected genes coding for resistance to tetracyclines and sulfonamides in the collected samples. The 16S rRNA gene was used as the reference gene to account for variability of total amount of bacteria in the samples. Primer sequences adapted from other studies [31,42,77,78] or designed for this study (*tetM*) are listed in Table 5. All qPCR assays were performed in the Roche LightCycler^®^ 480 II (Roche Applied Science, Indianapolis, IN, USA) in 11 μL reaction mixture. Analyzes for each sample were carried out in triplicate. PCR mixtures consisted of 5-μL LightCycler 480 SYBR Green I Master (Roche Applied Sciences, Indianapolis, IN, USA), 2.5 μL each primer of corresponding concentration 5 μM (*tetA, B, C, L, M, O, X,* *sulI*, *II*), 2 μM (*tetG, K, sulIII*), 1 μM (*tetQ*) and 1 μL DNA template of 5 ng/μL. In each run, 1 μL microbial DNA-free water as negative control was included. The thermal cycling conditions selected for studied genes are given in Table 6. Specificity of the amplified PCR product was verified by performing melting curve analysis, while amplification efficiency was assessed by monitoring the slope of amplification curves generated for the target genes and the internal controls (16S rRNA). Quantitative analysis of qPCR data was carried out using a comparative C_T_ method (also known as ΔΔC_T_, or 2^−ΔΔCT^) based on the literature [79]. In the first stage, threshold cycles (C_T_) of the tested ARGs and control gene (16S rRNA) amplification reactions in all samples were determined. The C_T_ value is provided as the result of qPCR analysis for each gene. In turn, for individual samples (raw influent and final effluent), the differences between the C_T_ values for the tested gene and 16S rRNA were calculated according to Formula (1). The obtained results present the ARG abundance relative to that of 16S rRNA. Then the ΔΔC_T_ values for each gene were obtained with the use of Formula (2). Computation the normalized value of the relative level of the tested gene in the unknown sample (final effluent) relative to the calibrator sample (raw influent) was carried out based on the Formula (3). The value of parameter R equal to 1 means the relative amount of gene in both samples is comparable, <1 indicates a decrease in the relative amount of the gene in the final wastewater sample, while >1 shows the enrichment of the gene after the wastewater treatment process.

ΔC_T (influent/effluent)_ = C_T (ARG)_ − C_T (16S rRNA)_ (1)

ΔΔCT = ΔCT_(effluent)_ − ΔCT_(influent)_ (2)

R = 2^−ΔΔCT^ (3)

## 4. Conclusions

A comparative quantitative analysis of 10 genes coding resistance to tetracycline and three sulfonamide resistance genes in two Polish WWTPs in most cases showed an enrichment of selected ARGs after the wastewater treatment processes. The results have confirmed that WWTPs are hotspots for the spread of antibiotic resistance determinants in the environment. This is a serious problem due to the introduction of final effluent into surface waters, as well as the widespread use of reclaimed wastewater as a fertilizer in agriculture soils. The results of this study highlight the need to implement effective actions to prevent the spread of antibiotic determinants in the environment, such as advanced wastewater treatment processes application, the implementation of permanent microbiological monitoring, taking into account the antibiotic resistance aspect, as well as increased control of drug intake and appropriate management of medical waste. Moreover, the specially targeted separate treatment of hospital wastewater before discharge to conventional WWTPs is an issue that needs to be addressed by adapting to local circumstances.

## Figures and Tables

**Table 1:** Results of quantitative and qualitative analysis of DNA extracted from activated sludge collected in WWTP1 using different commercially available DNA isolation and purification kits.

No.	DNA Extraction Kit	µg of Total DNA per 1 g of AS	A_260/280_	A_260/230_
1	Genomic Mini AX Stool (A&A Biotechnology, Gdynia, Poland)	117.5 ± 0.3	1.90 ± 0.02	1.77 ± 0.03
2	Genomic Mini AX Bacteria + (A&A Biotechnology, Gdynia, Poland)	274.6 ± 1.6	1.92 ± 0.01	1.78 ± 0.04
3	Exgene Soil DNA mini (GeneAll Biotechnology, Seoul, Korea)	365.4 ± 4.5	2.00 ± 0.01	1.31 ± 0.62
4	FastDNA SPIN Kit for Soil (MP Biomedicals, Solon, OH, USA)	751.8 ± 3.0	1.91 ± 0.11	0.55 ± 0.01
5	NucleoSpin Soil lysis buffer 1 (Macherey–Nagel, Düren, Germany)	297.9 ± 16.9	1.98 ± 0.01	1.93 ± 0.08
6	NucleoSpin Soil lysis buffer 2 (Macherey–Nagel, Düren, Germany)	306.2 ± 9.8	1.97 ± 0.01	1.97 ± 0.04
7	PowerSoil DNA Isolation Kit (Qiagen, Hilden, Germany)	21.4 ± 1.4	1.76 ± 0.22	1.02 ± 0.31
8	ZymoBIOMICS DNA Minikit (Zymo Research, Irvine, CA, USA)	247.9 ± 0.5	1.89 ± 0.02	1.81 ± 0.02
9	GeneMATRIX SOIL DNA Purification Kit (EURx, Gdańsk, Poland)	274.6 ± 1.6	1.92 ± 0.01	1.78 ± 0.04
10	GeneMATRIX Environmental DNA & RNA Purification Kit (EURx, Gdańsk, Poland)	213.5 ± 0.8	2.18 ± 0.01	2.15 ± 0.01

The above results are the mean values and standard deviations calculated for three replicates of each isolation.

**Table 2:** Results of comparative qPCR analysis for WWTP1.

ARGs	ARG Enrichment			
	Winter	Spring	Summer	Autumn
*tetA*	2.2	0.9	2.1	1.0
*tetB*	9.3	5.6	11.1	0.2
*tetC*	0.8	0.4	2.5	1.0
*tetG*	3.6	5.5	3.8	1.3
*tetK*	1.7	15.1	7.7	2.3
*tetL*	8.2	1.6	12.9	4.0
*tetM*	7.2	5.3	0.8	0.9
*tetO*	12.0	1.0	2.9	1.0
*tetQ*	0.7	4.0	4.2	0.6
*tetX*	0.6	2.2	2.2	2.5
*sulI*	0.9	1.2	6.9	0.9
*sulII*	0.9	1.5	7.7	0.9
*sulIII*	14.0	2.3	5.4	1.8

The above results are the enrichment factors (R) calculated for three replicates of each reaction.

**Table 3:** Results of comparative qPCR analysis for WWTP2.

ARGs	ARG Enrichment			
	Winter	Spring	Summer	Autumn
*tetA*	1.2	7.4	3.4	28.8
*tetB*	1.5	0.4	2.7	0.4
*tetC*	6.2	1.2	0.9	1.1
*tetG*	0.7	2.0	1.7	0.6
*tetK*	1.2	3.1	0.4	2.5
*tetL*	0.3	1.6	7.0	0.2
*tetM*	0.3	0.6	3.2	0.3
*tetO*	0.5	2.5	3.8	0.2
*tetQ*	3.9	2.0	1.1	0.9
*tetX*	6.8	0.9	1.3	1.6
*sulI*	3.1	1.3	1.0	1.1
*sulII*	1.1	1.1	7.2	0.5
*sulIII*	1.2	4.7	5.4	1.0

The above results are the enrichment factors (R) calculated for three replicates of each reaction.

**Table 4:** Main technological parameters of the studied WWTPs.

Parameter	Unit	WWTP1		WWTP2	
		Influent	Effluent	Influent	Effluent
BOD_5_	mgO_2_/L	446.0	3.7	449.0	2.6
COD	mgO_2_/L	1009.0	35.0	1066.0	32.5
TSS	mg/L	516.0	5.0	501.0	6.1
TP	mg/L	10.8	0.4	10.7	0.2
TN	mg/L	87.0	8.0	87.7	7.9

**Table 5:** PCR and qPCR amplification primers.

Target Gene	Primer Sequence (5′-3′)		Amplicon Size (bp)	Source
16S rRNA	F	CGG TGA ATA CGT TCY CGG	146	[77]
	R	GGW TAC CTT GTT ACG ACTT		
*tetA*	F	GCT ACA TCC TGC TTG CCT TC	210	[78]
	R	CAT AGA TCG CCG TGA AGA GG		
*tetB*	F	TAC GTG AAT TTA TTG CTT CGG	206	[42]
	R	ATA CAG CAT CCA AAG CGC AC		
*tetC*	F	CTT GAG AGC CTT CAA CCC AG	418	[78]
	R	ATG GTC GTC ATC TAC CTG CC		
*tetG*	F	GCT CGG TGG TAT CTC TGC TC	468	[78]
	R	AGC AAC AGA ATC GGG AAC AC		
*tetK*	F	TCG ATA GGA ACA GCA GTA	169	[78]
	R	CAG CAG ATC CTA CTC CTT		
*tetL*	F	TCG TTA GCG TGC TGT CAT TC	267	[78]
	R	GTA TCC CAC CAA TGT AGC CG		
*tetM*	F	AAT AAA TCA TAA ACA GAA AGC TTA TTA TAT AAC	171	This study
	R	AAT AAA TCA TAA TGG CGT GTC TAT GAT GTT CAC		
*tetO*	F	AAC TTA GGC ATT CTG GCT CAC	515	[78]
	R	TCC CAC TGT TCC ATA TCG TCA		
*tetQ*	F	AGA ATC TGC TGT TTG CCA GTG	169	[42]
	R	CGG AGT GTC AAT GAT ATT GCA		
*tetX*	F	CAA TAA TTG GTG GTG GAC CC	468	[78]
	R	TTC TTA CCT TGG ACA TCC CG		
*sulI*	F	GAC GAG ATT GTG CGG TTC TT	185	[31]
	R	GAG ACC AAT AGC GGA AGCC		
*sulII*	F	GAC AGT TAT CAA CCC GCG AC	147	[31]
	R	GTC TTG CAC CGA ATG CAT AA		
*sulIII*	F	ACC ACC GAT AGT TTT TCC GA	199	[31]
	R	TGC CTTT TTC TTT TAA AGCC		

**Table 6:** qPCR conditions used in this study.

Reaction Stage	*tet*(*B, K, L, M, Q, X*), *sulIII*		*tet*(*G*, *C*), *sulI*, *sulII*		*tet*(*A, O*)	
Pre-incubation	95 °C, 5 min (4.4 °C/s)		95 °C, 5 min (4.4 °C/s)		95 °C, 5 min (4.4 °C/s)	
Amplification	95 °C, 10 s (4.4 °C/s)	45 cycles	95 °C, 10 s (4.4 °C/s)	45 cycles	95 °C, 10 s (4.4 °C/s)	55 cycles
	62 °C, 30 s (2.2 °C/s)		60 °C, 30 s (2.2 °C/s)		60 °C, 30 s (2.2 °C/s)	
	72 °C, 30 s (4.4 °C/s)		72 °C, 30 s (4.4 °C/s)		72 °C, 30 s (4.4 °C/s)	
Melting curve	95 °C, 5 s (4.4 °C/s)		95 °C, 5 s (4.4 °C/s)		95 °C, 5 s (4.4 °C/s)	
	65 °C, 1 min (2.2 °C/s)		65 °C, 1 min (2.2 °C/s)		65 °C, 1 min (2.2 °C/s)	
